# High levels of anti-inflammatory and pro-resolving lipid mediators lipoxins and resolvins and declining docosahexaenoic acid levels in human milk during the first month of lactation

**DOI:** 10.1186/1476-511X-12-89

**Published:** 2013-06-15

**Authors:** Gisela Adrienne Weiss, Heinz Troxler, Glynis Klinke, Daniela Rogler, Christian Braegger, Martin Hersberger

**Affiliations:** 1 Clinical Chemistry and Biochemistry, Children’s Research Center, University Children’s Hospital Zurich, Zurich, Switzerland; 2 Zurich Center for Integrative Human Physiology, University of Zurich, Zurich, Switzerland; 3 Institute of Physiology, University of Zurich, Zurich, Switzerland; 4 Gastroenterology and Nutrition, University Children’s Hospital Zurich, Zurich, Switzerland

**Keywords:** Human milk, Omega-3 fatty acids, DHA, EPA, 17-HDHA, 18-HEPE, 15-HETE, Resolvins, Lipoxins, Leukotrienes

## Abstract

**Background:**

The fatty acid mixture of human milk is ideal for the newborn but little is known about its composition in the first few weeks of lactation. Of special interest are the levels of long-chain PUFAs (LCPUFAs), since these are essential for the newborn’s development. Additionally, the LCPUFAs arachidonic acid (AA), eicosapentaenoic acid (EPA) and docosahexaenoic acid (DHA) are precursors for lipid mediators which regulate inflammation.

**Methods:**

We determined the composition of 94 human milk samples from 30 mothers over the first month of lactation for fatty acids using GC-MS and quantified lipid mediators using HPLC-MS/MS.

**Results:**

Over the four weeks period, DHA levels decreased, while levels of γC18:3 and αC18:3 steadily increased. Intriguingly, we found high concentrations of lipid mediators and their hydroxy fatty acid precursors in human milk, including pro-inflammatory leukotriene B4 (LTB4) and anti-inflammatory and pro-resolving lipoxin A4 (LXA4), resolvin D1 (RvD1) and resolvin E1 (RvE1). Lipid mediator levels were stable with the exception of two direct precursors.

**Conclusions:**

Elevated levels of DHA right after birth might represent higher requirements of the newborn and the high content of anti-inflammatory and pro-resolving lipid mediators and their precursors may indicate their role in neonatal immunity and may be one of the reasons for the advantage of human milk over infant formula.

## Background

Human milk fat is the main energy source for the breast-fed newborn and provides specific fatty acids that are required for the newborn’s development. The fatty acid composition of human milk has been assessed in several studies showing differences between term and preterm milk [1-5] and the influence of diet on the fatty acid composition [6-8]. There is also indication for a change in fatty acid composition over the lactation period [5,9-18].

Especially, LCPUFAs fulfill several essential functions in the newborns. For example, the omega-6 LCPUFA AA and the omega-3 LCPUFA DHA are crucial for brain and nervous system development [19,20], the visual system [21] and for early human growth in general [22]. In addition, the fatty acid profile of human milk has been associated with the development of atopy and with allergic diseases in children [23-26].

The supply of LCPUFAs by human milk to the newborn determines the fatty acid composition of several lipid compartments including plasma lipids and the cellular membrane [27]. The fatty acid composition of the membrane influences not only membrane properties but also immune-regulatory processes through the metabolization of free and membrane bound LCPUFAs to lipid mediators [28]. These lipid mediators are signaling molecules that initiate and resolve inflammation and they derive from oxygenation of the omega-6 fatty acid AA and the two omega-3 fatty acids DHA and EPA [29,30]. Oxygenation of AA, DHA and EPA occurs in a concerted action of lipoxygenases, cyclooxygenases and cytochrome-P-450 dependent oxygenases to result in the pro-inflammatory leukotrienes and the anti-inflammatory lipoxins from AA and resolvins and protectins from DHA and EPA [29-34]. Apart from being anti-inflammatory, lipoxins, resolvins and protectins also initiate inflammation-resolving actions like recruitment of nonphlogistic monocytes and clearance of apoptotic polymorphonuclear neutrophils by macrophages [35-37]. Due to their potent immune-regulatory functions, these lipid mediators are thought to play a role in chronic inflammatory diseases like atherosclerosis, rheumatoid arthritis and inflammatory bowel disease [31,38-40]. We therefore surmised that a supply of anti-inflammatory and pro-resolving lipid mediators to the newborn by breast milk could be one of the explanations for the lower incidence of intestinal inflammation in breast-fed compared to formula-fed infants [41,42].

Only limited information is available on the fatty acid composition of human milk over the first few weeks of lactation [15-18], and the presence of leukotrienes, lipoxins and resolvins in human milk has not been investigated yet. In this study, we present the milk fatty acid profile and the profile for selected bioactive lipid mediators and their precursors in human milk over the first month of lactation.

## Results

### Quantification of fatty acid composition in human milk

**Figure 1:**
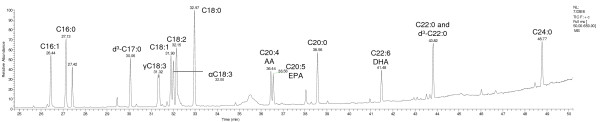
**Gas-chromatogram of fatty acid standard substances.** Fatty acids in standard mixture were methylated according to the sample preparation procedure prior to injection into the GC-MS system. For nomenclature of the fatty acids see Table 1.

Thirteen fatty acids containing 16 to 24 carbon atoms (Table 1 and Figure 1) were analysed and quantified in human milk with a specific and selective GC-MS method. For each analyte, the calibration was linear with R^2^ >0.98 and the observed fatty acid concentrations in breast milk were within the working range (See Additional file 1: Table S1). Intra-assay coefficients of variation are stated in (Additional file 1: Table S1).

**Table 1:** Fatty acids analyzed in human milk

**State of saturation**	**Fatty acid**	**Lipid number (*C*:*D*)**^**a**^
Saturated	Palmitic acid	C16:0
	Stearic acid	C18:0
	Arachidic acid	C20:0
	Behenic acid	C22:0
	Lignoceric acid	C24:0
Monounsaturated	Palmitoleic acid	C16:1
	Oleic acid	C18:1
Omega-6 polyunsaturated	Linoleic acid	C18:2
	γ-Linolenic acid	γC18:3
	Arachidonic acid	C20:4
Omega-3 polyunsaturated	α-Linolenic acid	αC18:3
	Eicosapentaenoic acid	C20:5
	Docosahexaenoic acid	C22:6

^a^*C*, number of carbon atoms; *D*, number of double bonds

### Fatty acid composition of human milk over the first month of lactation

**Figure 2:**
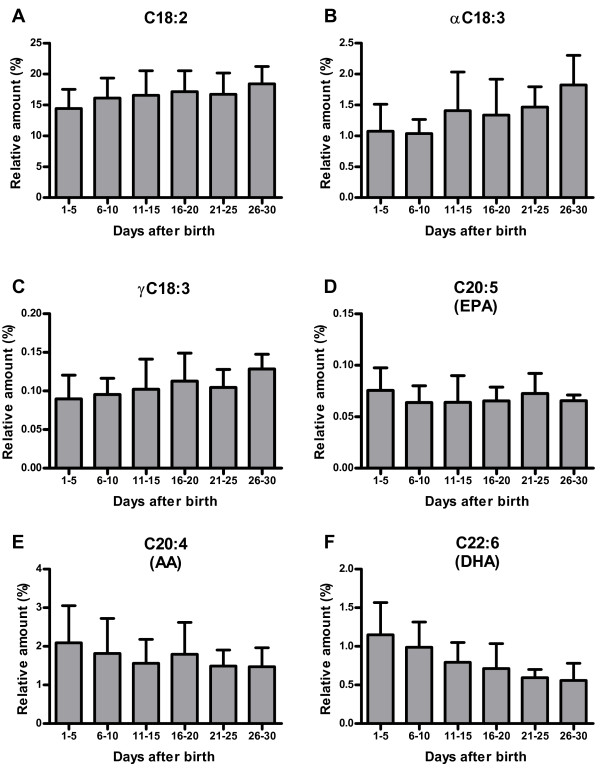
**Changes in omega-6 and omega-3 fatty acids in human milk over the first month of lactation.** Relative amount of the omega-6 PUFA (**E**) AA (C20:4) and its precursors (**A**) C18:2 and (**C**) γC18:3. Relative amount of the omega-3 PUFA (**F)** DHA (C22:6) and its precursors (**D**) αC18:3 and (**B**) EPA (C20:5). For an overview on PUFA fatty acid biosynthesis see Figure 3.

The lactational age of the collected human milk samples ranged from 1 to 30 days postpartum. For statistical analysis, the samples were divided into six groups according to their lactational age (Table 2). The major fatty acids in human milk were C18:1, C16:0, C18:2 and C18:0 in descending order. Other fatty acids were present with quantities lower than 5% of total fatty acids. The relative content was stable over the first month of lactation for the saturated fatty acids C16:0 to C22:0. Only C24:0 showed a significant decrease (Table 2). The amounts of the monounsaturated fatty acids C16:1 and C18:1 were stable over the observed time period. In contrast, the amount of most PUFAs changed over time (Figure 2). The amount of DHA decreased to approximately half of the initial quantity (p < 0.0001, Table 2), while its precursor αC18:3 increased over the same period. Similarly, the amount of AA showed a trend to decrease over time (p=0.094) while its precursor γC18:3 increased. Albeit these changes in the concentration of the PUFAs, the ratio of omega-6 (C18:2, γC18:3, AA) to omega-3 (αC18:3, EPA, DHA) fatty acids in human milk did not change significantly over the first four weeks of lactation.

**Table 2:** Total fatty acid composition (%) of human milk over time of lactation

**Fatty acid**	**Time of lactation (days after birth)**						**R**^**2**^	**P**
	**1-5**	**6-10**	**11-15**	**16-20**	**21-25**	**26-30**		
	**N=15**	**N=27**	**N=23**	**N=16**	**N=6**	**N=7**		
	**M**	**M**	**M**	**M**	**M**	**M**		
	**SD**	**SD**	**SD**	**SD**	**SD**	**SD**		
C16:0	23.96	20.75	21.96	21.95	21.90	21.92	0.12	0.63
	3.47	2.41	2.55	2.59	2.60	4.02		
C18:0	6.91	6.06	7.73	7.34	6.85	7.64	0.23	0.37
	1.59	1.31	1.90	1.69	0.67	1.36		
C20:0	0.17	0.11	0.15	0.14	0.12	0.12	0.36	0.10
	0.06	0.03	0.04	0.05	0.03	0.03		
C22:0	0.22	0.13	0.15	0.12	0.13	0.17	0.16	0.27
	0.18	0.04	0.06	0.04	0.04	0.03		
C24:0	0.29	0.17	0.20	0.16	0.14	0.18	0.48	0.011
	0.12	0.07	0.10	0.09	0.05	0.05		
C16:1	3.35	4.55	3.66	4.30	4.27	3.67	0.02	0.74
	0.87	1.35	1.17	1.81	1.21	0.89		
C18:1	46.29	48.21	45.76	44.92	46.26	43.96	0.46	0.51
	5.42	3.94	5.28	3.80	5.17	2.50		
C18:2	14.42	16.10	16.58	17.16	16.72	18.43	0.83	0.13
	3.09	3.24	3.97	3.37	3.45	2.80		
γC18:3	0.09	0.10	0.10	0.11	0.11	0.13	0.81	0.040
	0.03	0.02	0.04	0.04	0.02	0.02		
C20:4 (AA)	2.09	1.82	1.56	1.79	1.49	1.48	0.73	0.094
	0.96	0.91	0.62	0.83	0.41	0.49		
αC18:3	1.07	1.04	1.41	1.34	1.47	1.82	0.85	0.0024
	0.44	0.23	0.62	0.58	0.33	0.48		
C20:5 (EPA)	0.08	0.06	0.06	0.07	0.07	0.07	0.06	0.77
	0.02	0.02	0.03	0.01	0.02	0.01		
C22:6 (DHA)	1.15	0.99	0.79	0.71	0.59	0.56	0.96	<0.0001
	0.42	0.33	0.25	0.32	0.11	0.22		
n6/n3	7.58	8.92	8.31	9.47	8.81	8.27	0.12	0.59
	1.73	2.40	1.50	3.11	2.53	1.00		

M, mean % of total fatty acids; SD, standard deviation; R^2^, coefficient of determination of linear trendline; P, p-value for linear trend (ANOVA)

### Quantification of selected lipid mediators and precursors in human milk

**Figure 3:**
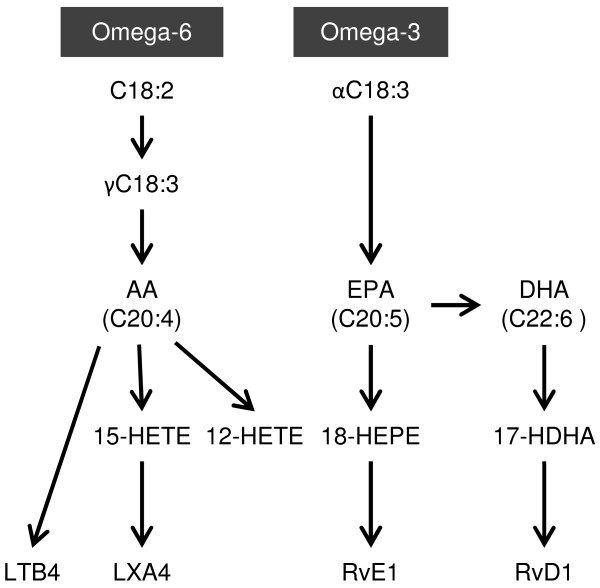
**Simplified scheme for the biosynthesis of the selected measured lipid mediators.** LTB4, LXA4, RvE1 and RvD1 are produced from the two essential omega-6 and omega-3 fatty acid pathways via the corresponding intermediate mono-hydroxy fatty acids.

**Figure 4:**
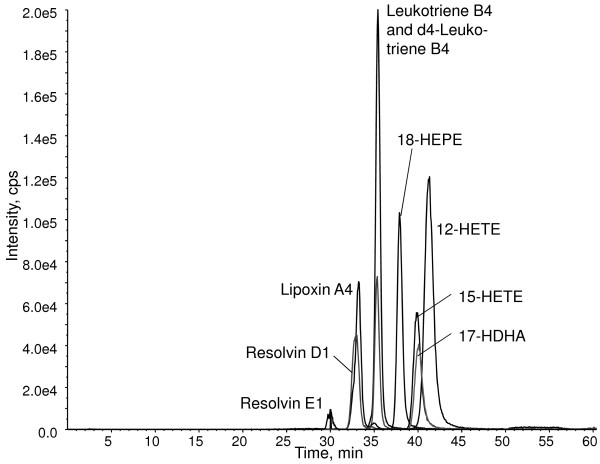
**Chromatogram of lipid mediators and related precursor hydroxy fatty acids.** 5 μl of a 0.1 ng/μl standard mixture was injected into the HPLC-MS/MS system. Cps, counts per second.

To determine the concentration of the selected lipid mediators in human breast milk, a HPLC-MS/MS method was developed analyzing LTB4, LXA4, RvE1 and RvD1 and the four hydroxy fatty acids 17-hydroxydocosahexaenoic acid (17-HDHA), 18-hydroxyeicosapentaenoic acid (18-HEPE), 15-hydroxyeicosatetraenoic acid (15-HETE) and 12-hydroxyeicosatetraenoic acid (12-HETE) (Figures 3 and 4). For each compound, several transitions were scanned (See Addtional file 1: Table S2) and one transition per analyte was selected as quantifier ion. For each lipid mediator and hydroxy fatty acid, the fragmentation pattern of the analyte from human milk was comparable to the fragmentation pattern of the corresponding standard substance (See Additional file 2: Figure S1, and Additional file 3: Figure S2). For each analyte, the calibration was linear with R^2^ >0.98 and the observed lipid mediator concentrations in breast milk were within the working range. Intra-assay coefficients of variation are stated in (Additional file 1: Table S2).

### Lipid mediator concentrations of human milk over the first month of lactation

**Figure 5:**
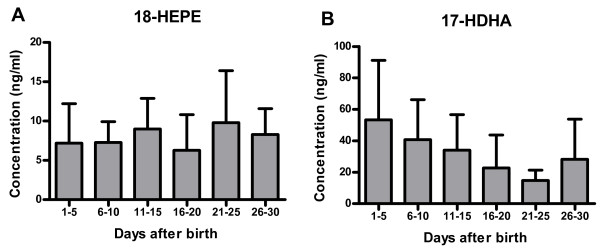
**Changes in the concentration of the precursor hydroxy fatty acids over the first month of lactation.** Concentrations of (**A**) 18-HEPE (precursor of RvE1) and (**B**) 17-HDHA (precursor of RvD1) were measured in human milk over the first month of lactation.

The breast milk concentration of the lipid mediators LTB4, LXA4, RvE1 and RvD1 showed mean values (and ranges) of 9 ng/ml (0.01 to 24.5 ng/ml) for LTB4, 21 ng/ml (0.1 to 54.1 ng/ml) for LXA4, 6 ng/ml (0.03 to 41.5 ng/ml) for RvE1 and 10 ng/ml (0.01 to 44.5 ng/ml) for RvD1, and was stable over the period of one month for all lipid mediators (Table 3). The mean concentrations of the precursor hydroxy fatty acids were higher than the concentrations of the more bioactive forms with the exception of 18-HEPE, which was observed at similar concentrations to the resulting lipid mediator RvE1 at the beginning of lactation. Over the observed four weeks of lactation, 18-HEPE increased in concentration, although not significantly (p=0.063). In contrast, the 17-HDHA content of human breast milk decreased. It was more than three times higher in the first five days postpartum than three weeks postpartum (Figure 5), which followed the change in DHA content of the human breast milk. The concentrations of 12-HETE and 15-HETE were stable over the observed time period.

**Table 3:** Concentration (ng/ml) of lipid mediators and precursors in human milk over time of lactation

**Compound**	**Time of lactation (days after birth)**						**R**^**2**^	**P**
	**1-5**	**6-10**	**11-15**	**16-20**	**21-25**	**26-30**		
	**N=11-14**	**N=20-23**	**N=20-21**	**N=13-14**	**N=6**	**N=6-7**		
	**M**	**M**	**M**	**M**	**M**	**M**		
	**SD**	**SD**	**SD**	**SD**	**SD**	**SD**		
LTB4	9.48	10.01	10.19	8.47	9.31	7.78	0.51	0.44
	8.59	4.43	5.28	6.31	5.64	3.34		
LXA4	15.55	25.02	22.11	16.65	19.53	22.04	0.02	0.19
	16.87	14.68	13.11	14.06	10.11	12.60		
RvE1	4.24	5.23	8.15	4.60	12.57	5.67	0.19	0.13
	5.76	3.91	11.45	4.08	15.20	5.65		
RvD1	9.42	12.46	12.37	6.48	9.53	9.99	0.08	0.58
	11.68	7.34	9.28	6.24	4.64	5.06		
17-HDHA	53.38	40.73	34.04	22.72	14.76	28.27	0.71	0.0035
	37.80	25.41	22.61	20.94	6.61	25.50		
18-HEPE	7.20	7.29	8.99	6.28	9.80	8.30	0.18	0.063
	5.02	2.63	3.92	4.54	6.60	3.29		
15-HETE	28.38	24.19	26.90	25.35	22.80	26.04	0.22	0.60
	16.44	12.86	11.72	17.85	8.33	19.65		
12-HETE	43.90	38.46	36.71	28.12	28.75	23.63	0.95	0.39
	26.61	29.11	15.99	19.37	15.80	11.55		

M, mean; SD, standard deviation; R^2^, coefficient of determination of linear trendline; P, p-value for linear trend (ANOVA)

## Discussion

In this study, we examined the fatty acid and the derived lipid mediator profiles of human breast milk over the first month of lactation. While most of the fatty acids showed stable concentrations over the four weeks of lactation, the concentrations of the omega-6 fatty acid AA and of the omega-3 fatty acid DHA decreased over time. Concomitantly, the amount of the precursor fatty acids for both of these very long-chain PUFAs increased. Human breast milk also contains a considerable amount of lipid mediators and its precursor hydroxy fatty acids. While we did not observe a change in concentration for the lipid mediators over the investigated lactation period, the DHA metabolite 17-HDHA decreased in parallel to the DHA concentration (See Additional file 1: Table S3).

The most surprising finding of this study was the high concentration of bioactive lipid mediators and their precursors in human milk. The levels of these analytes in human milk have not been reported so far. All of the investigated lipid mediators LTB4, LXA4, RvE1 and RvD1 were found at considerable concentrations, which were higher than the concentrations reported for plasma of healthy individuals. While human breast milk contains in average 8 ng/ml of the pro-inflammatory LTB4, the level for LTB4 was reported to be 100 times lower in plasma with concentrations of approximately 34 pg/ml [43]. Similarly, the concentration for the anti-inflammatory lipid mediators is higher in human breast milk. While breast milk contains in average 18 ng/ml LXA4, the plasma levels were reported to be 100 times lower ranging from 10 to 26 pg/ml [44]. Also, RvD1 levels are 100 times lower in plasma than in human breast milk with 31 pg/ml [45] and 9 ng/ml, respectively. The levels for RvE1 are 10 times higher in human breast milk than in plasma with 6 ng/ml and 0.1 to 0.4 ng/ml of RvE1, respectively [33]. There is also a 10–100 times increase in the concentration of the precursors for these lipid mediators. While we detected 12-HETE, 15-HETE, 18-HEPE and 17-HDHA mean concentrations in human breast milk in the range of 5–40 ng/ml, these hydroxy fatty acids were measured in the low nanomolar range in human whole blood with concentrations ranging from 0.1 to 4.10 ng/ml [45,46]. Thus, there seems to be an enrichment of the lipid mediators and its precursors in human breast milk, similar to the enrichment previously observed for some of the prostaglandins [47].

Our data also suggest a preferred accumulation of anti-inflammatory and pro-resolving lipid mediators in human milk as seen by the bioactive products of AA. This fatty acid can be converted into pro- and anti-inflammatory lipid mediators like LTB4 and LXA4, respectively [48]. In human milk, the average content of anti-inflammatory LXA4 is double as high as for the pro-inflammatory LTB4, while in human whole blood the concentration of LXA4 seems to be lower than the concentration of LTB4 [43,44]. In addition, there are high concentrations of the precursor hydroxy fatty acids for the anti-inflammatory and pro-resolving lipid mediators present in human breast milk. At least some of the precursors also have potent anti-inflammatory and pro-resolving effects, which may derive from a direct effect or through its metabolism to the lipid mediators.

Several studies have shown that the lipid mediators LXA4, RvE1 and RvD1 have potent anti-inflammatory and pro-resolving effects in experimental mouse models of intestinal colitis [49-53]. In addition, also 17-HDHA, the precursor for RvD1, was shown to reduce inflammation in dextran sulfate sodium induced colitis in mice [52]. It is therefore tempting to speculate that these high levels of lipid mediators and their precursors in human milk might affect the immune regulation of the gastrointestinal tract in the newborn. In this line, the concentrations measured in human breast milk for RvD1 and LXA4 meet the concentrations necessary to effect human leukocytes in an anti-inflammatory and pro-resolving manner [54,55]. For example, LXA4 and RvE1 were shown to suppress neutrophil infiltration and to promote phagocytosis of bacteria and apoptotic neutrophils by macrophages in the nanomolar range [33,35,56,57]. In similar concentrations, analogs of LXA4 were able to reduce pathogen-induced interleukin-8 production in intestinal [58] and bronchial human epithelial cells [59], which resulted in inhibited neutrophil recruitment, and thus in reduced inflammation. Hence, the measured breast milk concentrations of anti-inflammatory and pro-resolving lipid mediators are within the physiological range reported to inhibit inflammatory processes, and therefore, might lower inflammation in the newborn infant.

The concentrations of the measured lipid mediators did not change over the first month of lactation in contrast to the two precursor hydroxy fatty acids 17-HDHA and 18-HEPE. While the concentration of 18-HEPE slightly increases during lactation, the concentration of 17-HDHA decreases in parallel to the decrease of its precursor fatty acid DHA. The breast milk concentrations of the lipid mediator products RvE1 and RvD1 do not show the same temporal pattern, which is in agreement with the just in time generation of these inflammatory regulators during an acute inflammation [48]. Similarly, the concentrations of all the lipid mediators investigated in this study are constant in breast milk over the first month of lactation. Hence, an effect of degradation during storage can be excluded, since all samples were stored for the same time period. However, it is not clear why the intermediate precursor fatty acids investigated are present in such high concentrations in human breast milk. Possibly, these fatty acids are supplied as pre-metabolized lipid mediators for a final conversion to the more potent lipid mediators in the newborn’s intestine. Considering the high concentration of these anti-inflammatory and pro-resolving hydroxy fatty acids and lipid mediators in human breast milk, we presume a potential role in lowering the overall inflammation in breast-fed infants, suggesting a possible reason for the lower incidence of inflammatory diseases in breast-fed compared to formula-fed children [60-62]. In addition, these lipid mediators may be involved in gastrointestinal pain reception in newborns, since RvE1 and RvD1 have been shown to reduce inflammatory pain in mice [63].

The average fatty acid profile found in our study is similar to the fatty acid compositions reported before [1,5,7,14,23,64]. Considering the profile over the first month of lactation, the relative content in milk is stable for most fatty acids, while the concentration of specific LCPUFAs changes over this period.

Similar results were found in recent studies in which human milk with equal lactational age was analyzed and which presented the same decrease of DHA and AA over time, while the precursor fatty acids C18:2, γC18:3 and αC18:3 increased during the same period [6,14]. It was previously speculated that the increase in the precursors C18:2, γC18:3 and αC18:3 might compensate for the declining levels of DHA and AA in human breast milk, which may come from the depletion of the maternal DHA stores [65]. Alternatively, the high relative amounts of DHA and AA in the beginning of lactation may well be a mechanism to compensate for the lower absolute fat content of human breast milk in the beginning of lactation [64,66]. This may assure a stable supply of DHA and AA to the newborn suggesting a regulation of the fatty acid secretion by the mammary gland, which is not only dependent on the maternal DHA intake and plasma levels.

DHA and AA are important for the neonatal development. Especially DHA has a high abundance in the brain and retina where it is accumulated predominantly in the last trimester of pregnancy and during the first year after birth [67]. Before birth, most of the accumulated DHA is delivered from the mother by transplacental transfer [68]. After birth, human milk is the only exogenous source of DHA for the breast-fed child. The endogenous de novo production of the newborn and the DHA stores in adipose tissue alone are not sufficient to maintain DHA homeostasis [69]. Infants fed formula without DHA showed reduced DHA stores in their tissue six months after birth and showed only half the accumulation rate for DHA in the brain compared to breast-fed infants [70]. At the same time, breast-fed infants further increased their DHA stores also in non-brain tissues. Hence, the newborn’s need for DHA is eminent and elevated DHA levels in human milk right after birth may represent the high requirement for DHA at that time.

Preterm infants are born with a lower DHA status, because they did not complete intrauterine DHA accumulation [71]. The importance of DHA for the development is underlined by the higher DHA content of human preterm milk [2] and by the elevated activity of enzymes required for LCPUFA production in preterm infants [72]. This suggests that the fatty acid composition of human milk is part of the natural mechanism to regulate the infant’s DHA status.

DHA and AA are on the one hand vital for neuronal and visual functions, and on the other hand pivotal for the neonatal development of the immune system. Several studies confirmed that the DHA status is linked to various immune processes [73-77] and that several LCPUFAs have the potential to influence the immune system. For example, atopic sensitization of the newborn is associated to the α-linolenic acid and omega-3 fatty acid supply [78] and a higher tissue status of omega-3 fatty acids results in reduced inflammation and less tissue injury in a colitis model in mice [79]. The latter was shown in transgenic mice expressing *fat-1*, the enzyme responsible for the endogenous production of the omega-3 and omega-6 LCPUFAs DHA and AA from the essential PUFAs. As mentioned before, these LCPUFAs are the precursors of the lipid mediators and the metabolism of these LCPUFAs to the corresponding lipid mediators may play a role in the protective effect of the LCPUFAs in innate and adaptive immune responses [80-82]. Hence, our results suggest that human milk may be an important regulator of neonatal immunity by providing not only the precursors, but also the bioactive forms of these lipid mediators.

## Conclusions

We investigated human milk to identify components that are responsible for the beneficial effect of human milk on neonatal health. Our results confirm a considerable amount of DHA and AA in human milk with higher levels in the beginning of lactation. Since the nutrient composition of human milk reflects the ideal mixture to satisfy the newborn’s needs, this likely mirrors the neonatal DHA and AA requirements. Moreover, we demonstrate the presence of bioactive lipid mediators in human milk which affect atopy and inflammation and therefore influence neonatal immunity. Our results support an addition of DHA and AA to infant formula which is often done nowadays, but still is not standard practice. The high content of anti-inflammatory lipid mediators and its precursors in human breast milk may indicate the crucial role of lipid mediators in neonatal immunity and may be a reason for the advantage of human breast milk over infant formula.

## Methods

### Sample collection and preparation for fatty acid analysis

94 human milk samples were obtained from 30 mothers and the lactational age was recorded. Samples were stored at −20°C for approximately 120 days. Sample preparation for total fatty acid analysis was essentially done according to Moser et al. [83]. 125 μl of human milk was diluted 1:2 with ultrapure water (ELGA Purelab Ultra, Labtec Service AG, Wohlen, Switzerland) and subjected to protein precipitation with 1 ml methanol-methylene chloride (3:1, v/v). 20 μg d_3_-C17:0 (Cambridge Isotope Laboratories, Inc., Andover, MA, USA) and 0.8 μg d_3_-C22:0 (Dr. Ehrenstorfer GmbH, Augsburg, Germany) were added as internal standards. Fatty acid methyl esters were prepared by the addition of 200 μl acetyl chloride and subsequent incubation at 75°C for one hour. The reaction was neutralized with 4 ml 7% K_2_CO_3_, and fatty acid methyl esters were extracted with 2 ml hexane. After centrifugation at 2500 rpm for 20 min, 1.6 ml of the hexane layer was dried under nitrogen and redissolved in 280 μl heptane for injection into the GC-MS system. Calibration curves were obtained with defatted cow’s milk spiked with known concentrations of fatty acid standards, thereby defining the individual working range for each fatty acid (See Additional file 1: Table S1).

### GC-MS of fatty acid methyl esters

One μl sample was injected into a Finnigan PolarisQ ion trap GC-MS system (Thermo Quest, Austin, TX, USA). The injector temperature was 280°C and fatty acid methyl esters were separated on a 30 m BGB-1701 column (BGB Analytik AG, Boeckten, Switzerland). The initial oven temperature of 50°C was hold for 8 min and then increased gradually by 5°C/min reaching a final temperature of 280°C. The ion source temperature and transfer line temperature was 230°C and 300°C, respectively. Analytes were detected as positive ions in full scan mode from 50 to 650 m/z. Specific mass traces were extracted for the quantification of each analyte (See Additional file 1: Table S1). Fatty acids were identified by comparison of retention time and mass spectrum with authentic standards.

### Sample preparation for analysis of lipid mediators and their precursors

85 of the human milk samples were stored at −20°C for approximately 330 days. The analyte extraction method was adapted from Yang et al. [84]. Two volumes of ice cold methanol and 3 ng of deuterated LTB4 (d_4_-LTB4; Enzo Life Sciences AG, Lausen, Switzerland) as internal standard were added to 1 ml human milk. The samples were centrifuged for 20 min at 2500 rpm and the supernatant was diluted with 5 ml ultrapure water. The diluted supernatant was loaded on a C18 solid phase extraction (SPE) column (Grace, Deerfield, IL, USA) prewashed with 5 ml 90% methanol and 5 ml 5% methanol. After sample loading, the SPE column was washed with 2x5 ml 5% methanol and the analytes were eluted with 3 ml 90% methanol. The eluate was dried under nitrogen and redissolved in 100 μl 35% methanol (2 mM ammonium acetate). The sample was filtered with a 0.2 μm syringe filter (BGB Analytik AG, Boeckten, Switzerland) and 5 μl were injected into a HPLC-MS/MS system. Calibration curves were obtained by lipid mediator standard addition to human milk at the concentrations 0, 15, 30, 45, 60, 75 and 90 ng/ml.

### HPLC-MS/MS of lipid mediators and hydroxy fatty acids

Analytes were separated using a HPLC system (UltiMate® HPLC, LC Packings, Dionex, Olten, Switzerland) with a C18 column (Luna 3u C18(2) 100A, 150x0.3 mm; Phenomenex, Brechbühler AG, Schlieren, Switzerland). A gradient was run over 10 min from 35% to 80% methanol (2 mM ammonium acetate) and was kept constant for 25 min at a flow rate of 4 μl/min. The column was rinsed with 100% methanol and equilibrated with 35% methanol (2 mM ammonium acetate). Analytes of interest were detected on a Sciex 4000 QTRAP mass spectrometer (AB SCIEX GmbH, Zug, Switzerland) in the negative ion mode. Several transitions per analyte were scanned in multiple reaction monitoring mode and are stated in (Additional file 1: Table S2). The transitions used for quantification were 335→195 for LTB4, 351→115 for LXA4, 349→195 for RvE1, 375→233 for RvD1, 343→281 for 17-HDHA, 317→167 for 18-HEPE, 319→175 for 15-HETE, 319→179 for 12-HETE and 339→197 for d_4_-LTB4. Fragments scanned for the bioactive anti-inflammatory and pro-resolving LXA4, RvE1 and RvD1 were in agreement with prominent product ions reported earlier [33,84-86]. The optimized MS parameters were defined as: Electrospray ionization (ESI), curtain gas (CUR) = 10, nebulizer gas (GS1) = 20, auxiliary gas (GS2) = 0, ionspray voltage (IS) = −4500 V, collision gas (CAD) = medium, entrance potential (EP) = −10 V, cell exit potential = −11 to −5 V, dwell time = 100 msec. Declustering potential (DP) and collision energy (CE) were optimized individually for each analyte (See Additional file 1: Table S2). Fragmentation patterns for each analyte as standard substance as well as isolated from human milk were obtained by product ion scans at MS parameter settings defined above.

### Data analysis

Statistical analysis was done using Microsoft Office Excel. To estimate the difference between groups, analysis of variance (ANOVA) with subsequent trend analysis was used. All values are presented as mean with standard deviation or as indicated. Two-sided p-values < 0.05 were considered significant.

## Abbreviations

12-HETE: 12-hydroxyeicosatetraenoic acid; 15-HETE: 15-hydroxyeicosatetraenoic acid; 17-HDHA: 17-hydroxydocosahexaenoic acid; 18-HEPE: 18-hydroxyeicosapentaenoic acid; AA: Arachidonic acid; DHA: Docosahexaenoic acid; EPA: Eicosapentaenoic acid; GC-MS: Gas chromatography mass spectrometry; HPLC-MS/MS: High-performance liquid chromatography tandem mass spectrometry; LCPUFA: Long-chain polyunsaturated fatty acid; LTB4: Leukotriene B4; LXA4: Lipoxin A4; RvD1: Resolvin D1; RvE1: Resolvin E1; SPE: Solid phase extraction.

## Competing interests

The authors declare that they have no competing interests.

## Authors’ contributions

GW, DR, CB, MH designed the study and DR and CB collected the human milk samples. GW, HT and GK developed and performed the GC-MS and the LC-MS/MS assays to determine the fatty acid and lipid mediator levels in human milk. GW, HT, GK and MH analyzed the data, and GW and MH wrote the manuscript. All authors read and approved the manuscript.

## Supplementary Material

Additional file 1: Table S1 Extracted mass traces and working range for fatty acid analysis. **Table S2** Scanned transitions for lipid mediator analysis. **Table S3** Fatty acid precursors (% of total fatty acids) and concentration (ng/ml) of corresponding hydroxy fatty acids and lipid mediators in human milk over time of lactation. Click here for file

Additional file 2: Figure S1 Fragmentation spectra of lipid mediator standard substances and lipid mediators from human milk. Spectra were obtained as product ion scans at collision energy specified in, Additional file 1: Table S2 Quantifier and qualifier product ions selected for each analyte are given in Additional file 1: Table S2 (A) Fragmentation of LTB4 standard and (B) LTB4 from human milk as products of 335 m/z; (C) Fragmentation of LXA4 standard and (D) LXA4 from human milk as products of 351 m/z; (E) Fragmentation of RvE1 standard and (F) RvE1 from human milk as products of 349; (G) Fragmentation of RvD1 standard and (H) RvD1 from human milk as products of 375. Rel. Int., Relative Intensity; m/z, mass per charge; Da, Dalton. Click here for file

Additional file 3: Figure S2 Fragmentation spectra of hydroxy fatty acid standard substances and hydroxy fatty acids from human milk. Spectra were obtained as product ion scans at collision energy specified in, Additional file 1: Table S2 Quantifier and qualifier product ions selected for each analyte are given in, Additional file 1: Table S2 (A) Fragmentation of 12-HETE standard and (B) 12-HETE from human milk as products of 319 m/z; (C) Fragmentation of 15-HETE standard and (D) 15-HETE from human milk as products of 319 m/z; (E) Fragmentation of 18-HEPE standard and (F) 18-HEPE from human milk as products of 317; (G) Fragmentation of 17-HDHA standard and (H) 17-HDHA from human milk as products of 343. Rel. Int., Relative Intensity; m/z, mass per charge; Da, Dalton. Click here for file
