# Supplementary material for: High levels of anti-inflammatory and pro-resolving lipid mediators lipoxins and resolvins and declining docosahexaenoic acid levels in human milk during the first month of lactation
Source: Lipids Health Dis. 2013 Jun 15;12:89. doi: 10.1186/1476-511X-12-89 (PMC3698171; doi:10.1186/1476-511X-12-89)
Supplement: Additional file 1: Table S1 — Extracted mass traces and working range for fatty acid analysis. Table S2 Scanned transitions for lipid mediator analysis. Table S3 Fatty acid precursors (% of total fatty acids) and concentration (ng/ml) of corresponding hydroxy fatty acids and lipid mediators in human milk over time of lactation. [file 1476-511X-12-89-S1.docx]

| **Supplementary table I:** Extracted mass traces and working range for fatty acid analysis | | | | |  |
| --- | --- | --- | --- | --- | --- |
| Fatty acid | Lipid number (*C*:*D*)^a^ | Extracted mass traces (m/z) | Working range (µg/ml) | Intra-assay coefficient of variation (%)  N=6 | |
| Palmitic acid | C16:0 | 87, 143, 270 | 1.2 - 2200 | 8.0 | |
| Stearic acid | C18:0 | 143, 199, 298 | 2.5 - 1200 | 12.6 | |
| Arachidic acid | C20:0 | 143, 283, 326 | 2.3 - 34 | 10.5 | |
| Behenic acid | C22:0 | 311, 354 | 3.5 - 60 | 20.2 | |
| Lignoceric acid | C24:0 | 143, 199, 382 | 3.4 - 75 | 16.8 | |
| Palmitoleic acid | C16:1 | 67, 81, 96 | 5.2 - 250 | 3.4 | |
| Oleic acid | C18:1 | 166, 222, 264 | 7.4 - 4000 | 2.6 | |
| Linoleic acid | C18:2 | 164, 220, 262 | 8.1 - 3000 | 1.9 | |
| γ-Linolenic acid | γC18:3 | 79, 292 | 0.1 - 16 | 12.4 | |
| Arachidonic acid | C20:4 | 79, 91 | 4.5 - 250 | 7.0 | |
| α-Linolenic acid | αC18:3 | 79, 91 | 1.9 - 180 | 15.2 | |
| Eicosapentaenoic acid | C20:5 | 79, 91, 384.5 | 1.7 - 16 | 12.2 | |
| Docosahexaenoic acid | C22:6 | 79, 91 | 7.1 - 120 | 13.0 | |
| d_3_-Margaric acid  d_3_-Behenic acid | d_3_-C17:0  d_3_-C22:0 | 188, 244, 287  202, 216, 258, 314, 357 |  |  | |
| ^a^ *C*, number of carbon atoms; *D*, number of double bonds | | | | | |

| **Supplementary table II:** Scanned transitions for lipid mediator analysis | | | |  |
| --- | --- | --- | --- | --- |
| Compound | Scanned transition (m/z) | Collision Energy (eV) | Declustering potential (V) | Intra-assay coefficient of variation (%)  N= 6 or 7 |
| LTB4 | 335→317 335→195 (Quantifier ion) | -24 -24 | -85 -85 | 6.0 |
| LXA4 | 351→235 351→217 351→115 (Quantifier ion) | -28 -20 -20 | -30 -30 -30 | 4.2 |
| RvE1 | 349→331 349→205 349→195 (Quantifier ion) 349→161 | -20 -24 -24 -24 | -30 -30 -30 -30 | 17.5 |
| RvD1 | 375→277 375→233 (Quantifier ion) 375→215 | -18 -18 -18 | -60 -60 -60 | 11.0 |
| 17-HDHA | 343→281 (Quantifier ion) 343→245 343→201 | -20 -20 -20 | -60 -60 -60 | 9.9 |
| 18-HEPE | 317→255 317→195 317→167 (Quantifier ion) | -25 -25 -25 | -60 -60 -60 | 7.8 |
| 15-HETE | 319→257 319→219 319→175 (Quantifier ion) | -27 -27 -27 | -80 -80 -80 | 21.4 |
| 12-HETE | 319→301 319→257 319→179 (Quantifier ion) | -23 -23 -23 | -80 -80 -80 | 18.6 |
| d_4_-LTB4 (Internal Standard) | 339→277 339→197 (Quantifier ion) | -20 -20 | -60 -60 |  |

| **Supplementary table III:** Fatty acid precursors (% of total fatty acids) and concentration (ng/ml) of corresponding hydroxy fatty acids and lipid mediators in human milk over time of lactation | | | | | | | | |
| --- | --- | --- | --- | --- | --- | --- | --- | --- |
| Compound | Time of lactation (days after birth) | | | | | | R² | P |
|  | 1-5  N=11-15 | 6-10  N=20-27 | 11-15  N=20-23 | 16-20  N=13-16 | 21-25  N=6 | 26-30  N=6-7 |  |  |
|  | M  SD | M  SD | M  SD | M  SD | M  SD | M  SD |  |  |
| C20:4 (AA) (%) | 2.09 | 1.82 | 1.56 | 1.79 | 1.49 | 1.48 | 0.73 | 0.094 |
|  | 0.96 | 0.91 | 0.62 | 0.83 | 0.41 | 0.49 |  |  |
| LTB4 (ng/ml) | 9.48 | 10.01 | 10.19 | 8.47 | 9.31 | 7.78 | 0.51 | 0.44 |
|  | 8.59 | 4.43 | 5.28 | 6.31 | 5.64 | 3.34 |  |  |
| 15-HETE (ng/ml) | 28.38 | 24.19 | 26.90 | 25.35 | 22.80 | 26.04 | 0.22 | 0.60 |
|  | 16.44 | 12.86 | 11.72 | 17.85 | 8.33 | 19.65 |  |  |
| LXA4 (ng/ml) | 15.55 | 25.02 | 22.11 | 16.65 | 19.53 | 22.04 | 0.02 | 0.19 |
|  | 16.87 | 14.68 | 13.11 | 14.06 | 10.11 | 12.60 |  |  |
| C22:6 (DHA) (%) | 1.15 | 0.99 | 0.79 | 0.71 | 0.59 | 0.56 | 0.96 | <0.0001 |
|  | 0.42 | 0.33 | 0.25 | 0.32 | 0.11 | 0.22 |  |  |
| 17-HDHA (ng/ml) | 53.38 | 40.73 | 34.04 | 22.72 | 14.76 | 28.27 | 0.71 | 0.0035 |
|  | 37.80 | 25.41 | 22.61 | 20.94 | 6.61 | 25.50 |  |  |
| RvD1 (ng/ml) | 9.42 | 12.46 | 12.37 | 6.48 | 9.53 | 9.99 | 0.08 | 0.58 |
|  | 11.68 | 7.34 | 9.28 | 6.24 | 4.64 | 5.06 |  |  |
| C20:5 (EPA) (%) | 0.08 | 0.06 | 0.06 | 0.07 | 0.07 | 0.07 | 0.06 | 0.77 |
|  | 0.02 | 0.02 | 0.03 | 0.01 | 0.02 | 0.01 |  |  |
| 18-HEPE (ng/ml) | 7.20 | 7.29 | 8.99 | 6.28 | 9.80 | 8.30 | 0.18 | 0.063 |
|  | 5.02 | 2.63 | 3.92 | 4.54 | 6.60 | 3.29 |  |  |
| RvE1 (ng/ml) | 4.24 | 5.23 | 8.15 | 4.60 | 12.57 | 5.67 | 0.19 | 0.13 |
|  | 5.76 | 3.91 | 11.45 | 4.08 | 15.20 | 5.65 |  |  |
