# Supplementary figures and images for: High levels of anti-inflammatory and pro-resolving lipid mediators lipoxins and resolvins and declining docosahexaenoic acid levels in human milk during the first month of lactation
Source: Lipids Health Dis. 2013 Jun 15;12:89. doi: 10.1186/1476-511X-12-89 (PMC3698171; doi:10.1186/1476-511X-12-89)

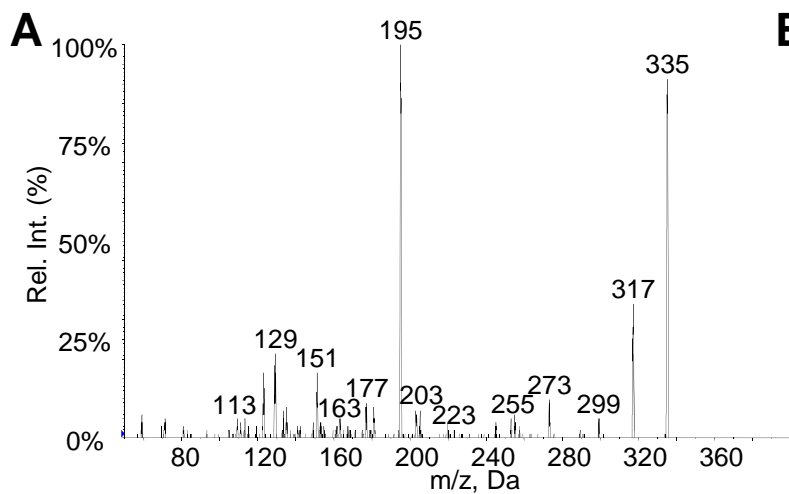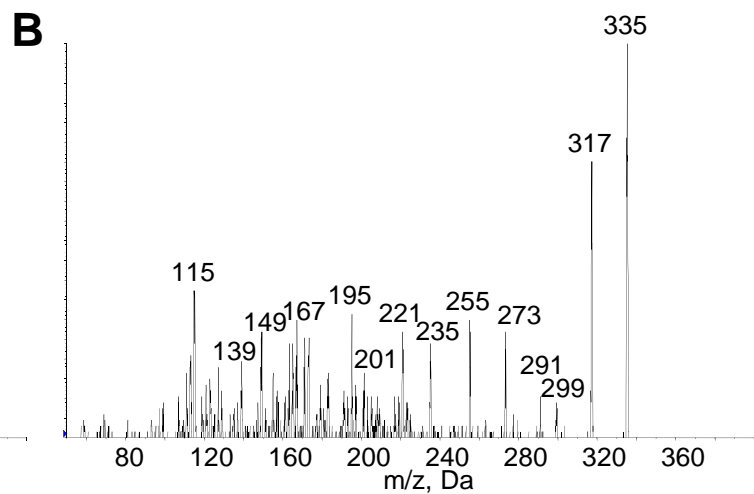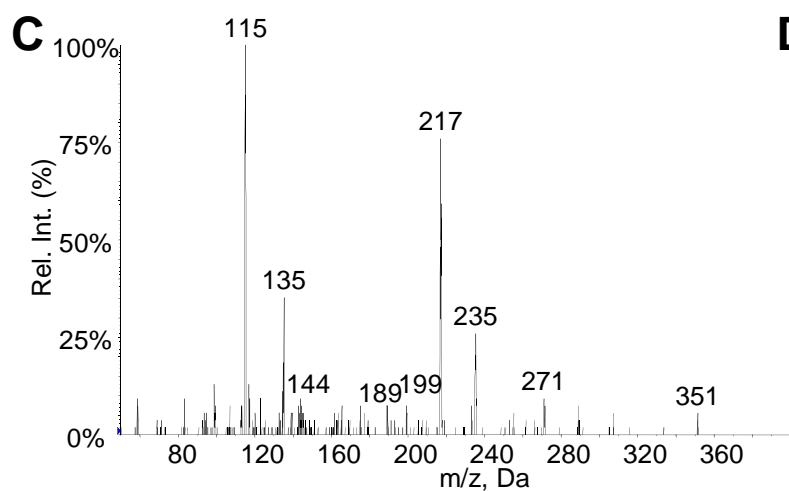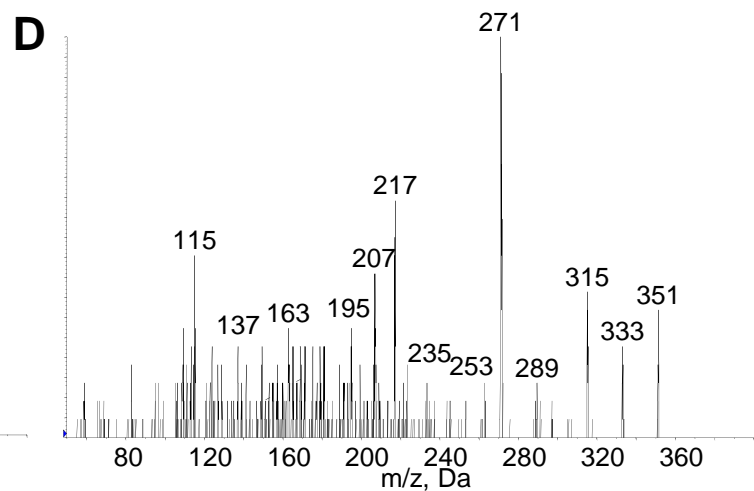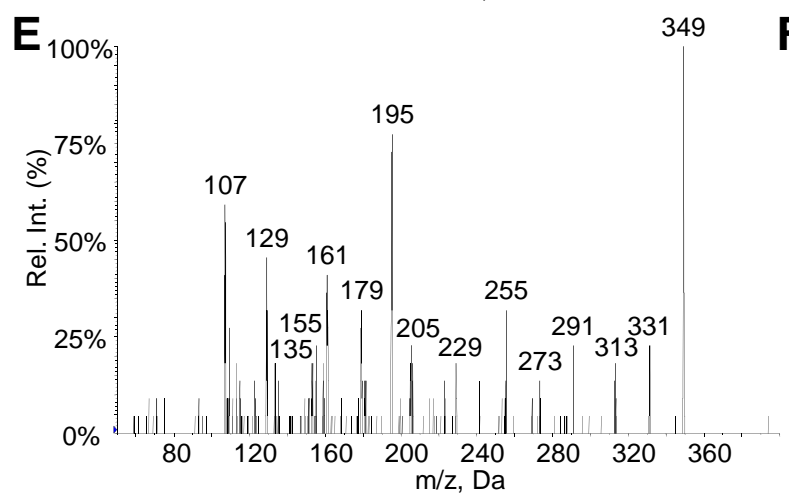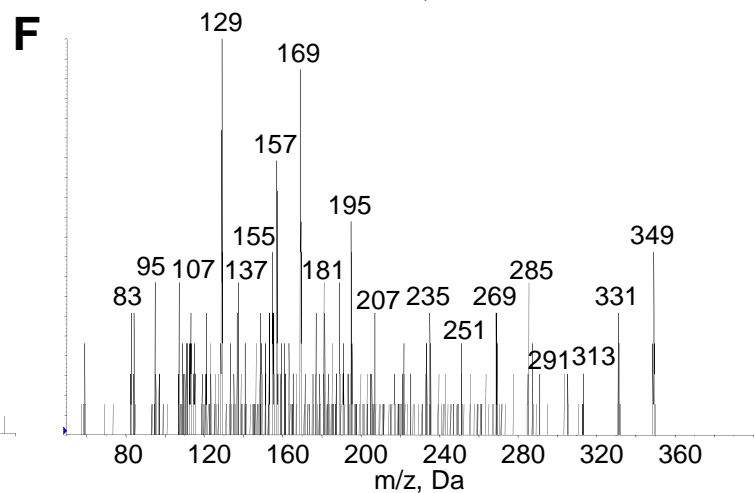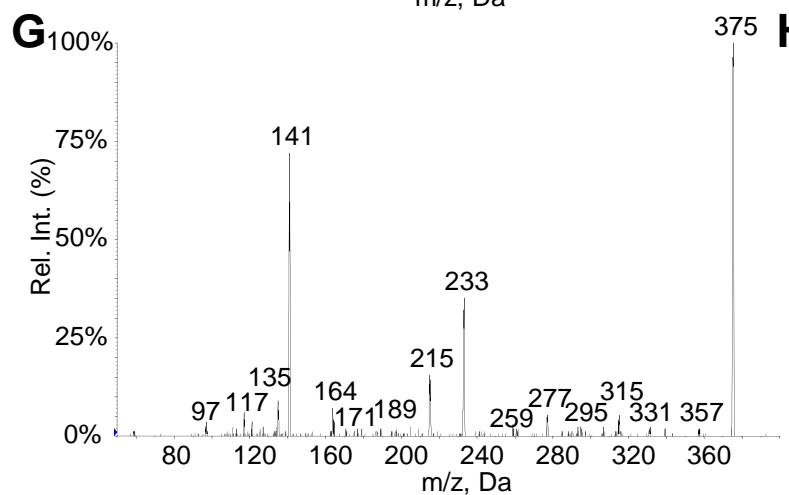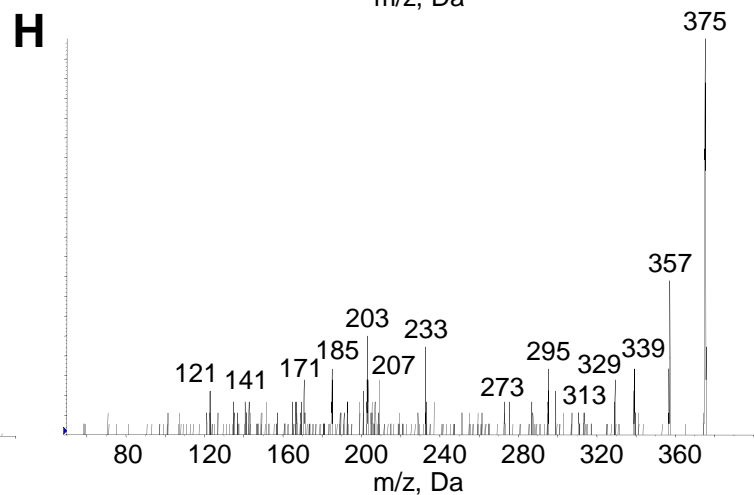

Supplement: Additional file 2: Figure S1 — Fragmentation spectra of lipid mediator standard substances and lipid mediators from human milk. Spectra were obtained as product ion scans at collision energy specified in, Additional file 1: Table S2 Quantifier and qualifier product ions selected for each analyte are given in Additional file 1: Table S2 (A) Fragmentation of LTB4 standard and (B) LTB4 from human milk as products of 335 m/z; (C) Fragmentation of LXA4 standard and (D) LXA4 from human milk as products of 351 m/z; (E) Fragmentation of RvE1 standard and (F) RvE1 from human milk as products of 349; (G) Fragmentation of RvD1 standard and (H) RvD1 from human milk as products of 375. Rel. Int., Relative Intensity; m/z, mass per charge; Da, Dalton. [file 1476-511X-12-89-S2.pdf]

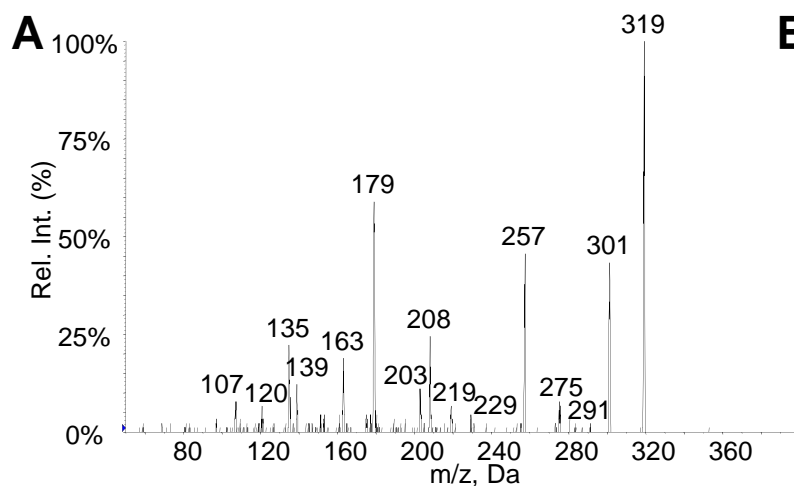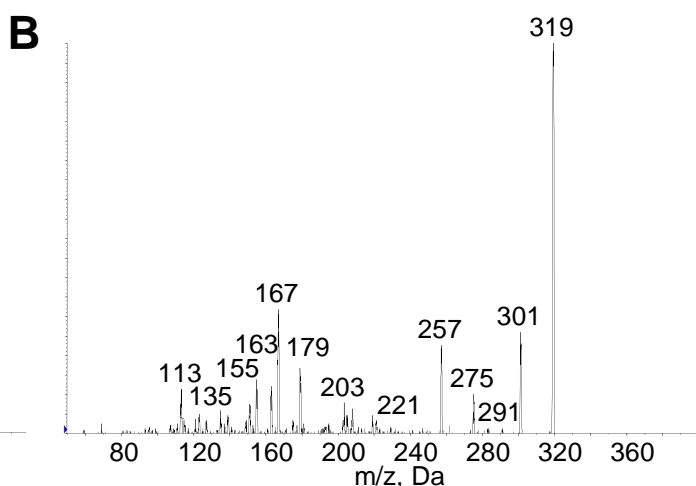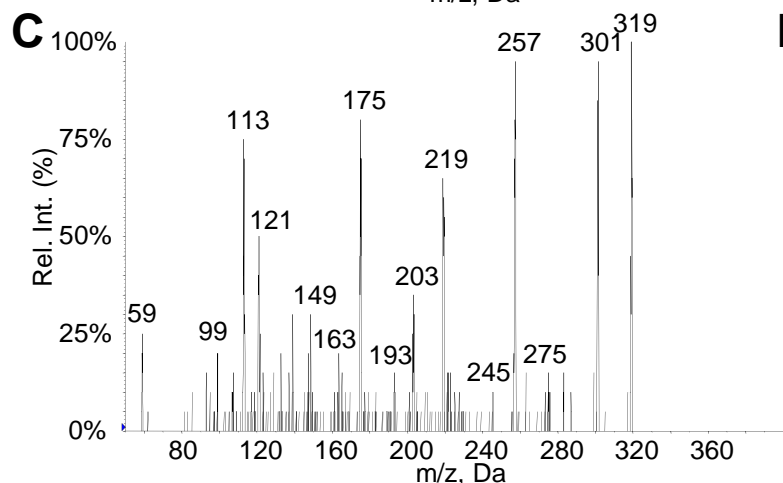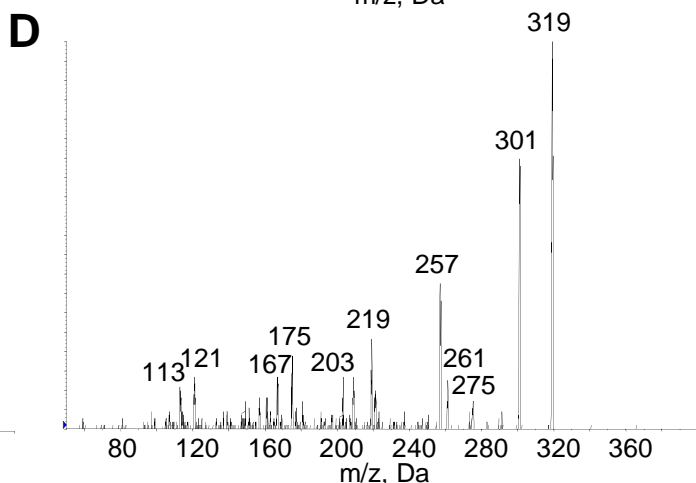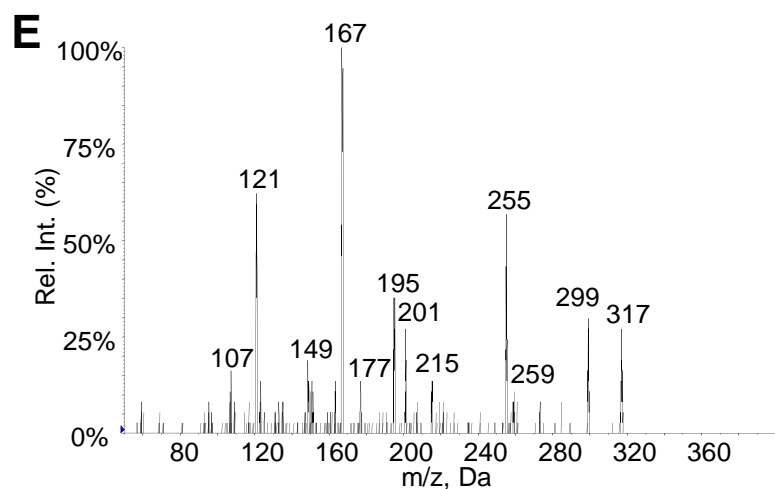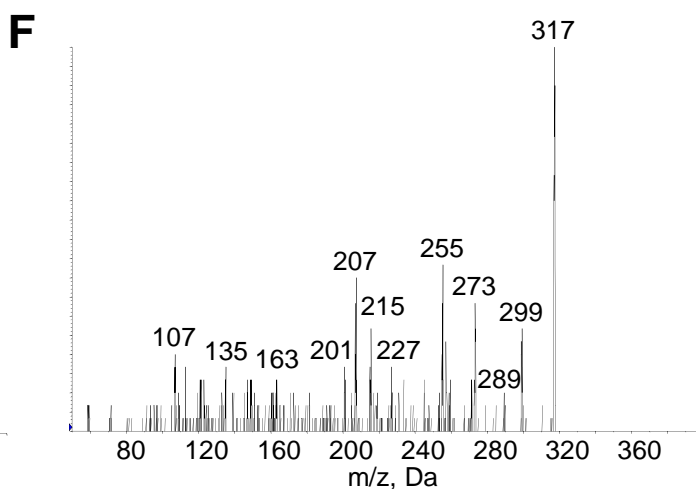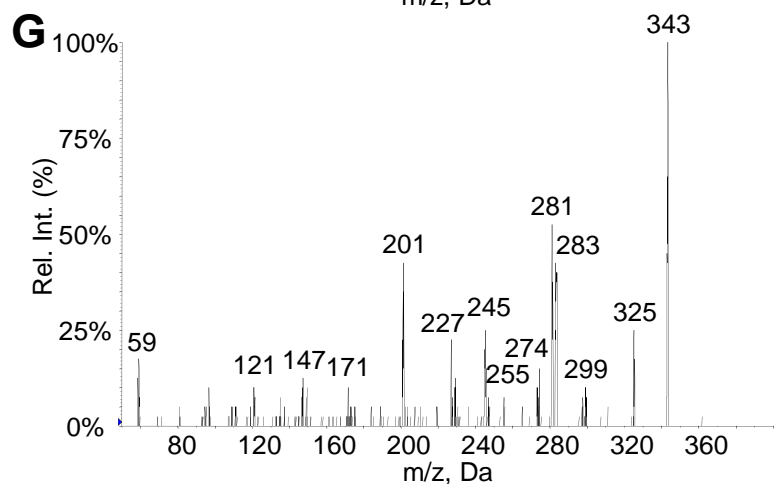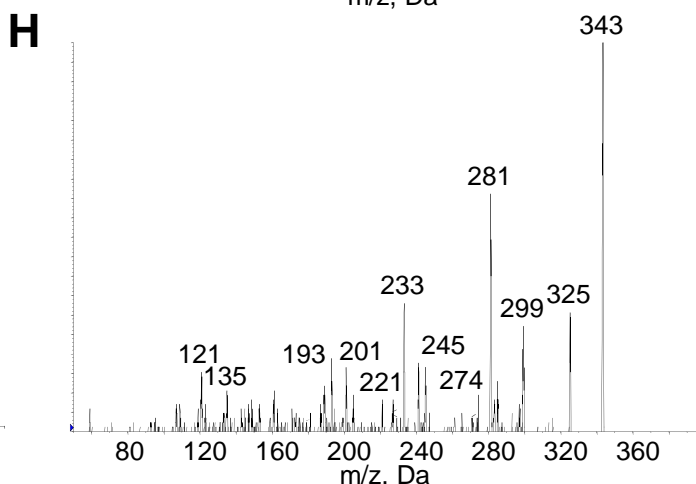

Supplement: Additional file 3: Figure S2 — Fragmentation spectra of hydroxy fatty acid standard substances and hydroxy fatty acids from human milk. Spectra were obtained as product ion scans at collision energy specified in, Additional file 1: Table S2 Quantifier and qualifier product ions selected for each analyte are given in, Additional file 1: Table S2 (A) Fragmentation of 12-HETE standard and (B) 12-HETE from human milk as products of 319 m/z; (C) Fragmentation of 15-HETE standard and (D) 15-HETE from human milk as products of 319 m/z; (E) Fragmentation of 18-HEPE standard and (F) 18-HEPE from human milk as products of 317; (G) Fragmentation of 17-HDHA standard and (H) 17-HDHA from human milk as products of 343. Rel. Int., Relative Intensity; m/z, mass per charge; Da, Dalton. [file 1476-511X-12-89-S3.pdf]
